# Optic Nerve Head Pulsatile Displacement in Open-Angle Glaucoma after Intraocular Pressure Reduction Measured by Optical Coherence Tomography: A Pilot Study

**DOI:** 10.3390/bioengineering11050411

**Published:** 2024-04-23

**Authors:** Marissé Masís Solano, Emmanuelle Richer, Santiago Costantino, Mark R. Lesk

**Affiliations:** 1 Maisonneuve-Rosemont Hospital Research Center, 5415 Assumption Blvd, Montreal, QC H1T 2M4, Canada; 2 Department of Ophthalmology, Université de Montréal, 5415 Assumption Blvd, Montreal, QC H1T 2M4, Canada; 3 École Polytechnique de Montréal, 2500 Chemin de Polytechnique, Montreal, QC H3T 1J4, Canada

**Keywords:** ocular biomechanics, optic nerve head displacement, optical coherence tomography pulsatility

## Abstract

This study investigated the effect of intraocular pressure (IOP) reduction on pulsatile displacement within the optic nerve head (ONH) in primary open-angle glaucoma (POAG) patients with and without axial myopia. Forty-one POAG patients (19 without myopia, 9 with axial myopia and 13 glaucoma with no intervention) participated. Swept-source optical coherence tomography (OCT) videos of the ONH were obtained before and after IOP-lowering treatment (medical or surgical) achieving a minimum IOP drop of 3 mmHg. A demons registration-based algorithm measured local pulsatile displacement maps within the ONH. Results demonstrated a significant 14% decrease in pulsatile tissue displacement in the non-myopic glaucoma cohort after intervention (*p* = 0.03). However, glaucoma patients with axial myopia exhibited no statistically significant change. There were no significant changes in the pulsatile ONH deformation in the control group. These findings suggest a potential link between IOP reduction and reduced pulsatile displacement within the ONH in POAG patients without myopia, offering new insights into the disease’s pathophysiology and warranting further investigation into underlying mechanisms and clinical implications.

## 1. Introduction

The optic nerve head (ONH) is the primary site of damage in glaucoma [1,2,3]. Intraocular pressure (IOP) is widely recognized as a major risk factor [4,5,6]. However, the complex interplay between IOP, ONH biomechanics, and disease progression remains poorly understood [7,8,9,10]. This lack of understanding underscores the need to investigate factors beyond IOP, particularly in light of individual variations due to age, demographics, and anatomy [11,12]. Furthermore, a recent publication based on the ocular hypertension treatment study (OHTS), a landmark glaucoma clinical trial, found that only 25% of the study participants with elevated IOP developed visual field loss in either eye over a 20-year follow-up [13]. The elusive nature of the pathophysiological pathways of POAG has led researchers to investigate the mechanical properties of the eye as prognostic biomarkers and potentially actionable targets.

Deformation of the lamina cribrosa (LC) and adjacent structures at the optic nerve head (ONH) have been investigated recently through analyses of physical changes during intraocular pressure (IOP) manipulation and gaze shift [14,15]. Studies on the response of the lamina cribrosa to IOP manipulation have yielded variable results [2,16,17], which may reflect the baseline differences and remodeling that occur in POAG. Currently, assessment of morphological changes at the ONH, both in animal and human studies [6,14,18,19], employ OCT technology. Various methods have been used, including 3D volume reconstruction [2], digital volume correlation algortitm [17], and in silico simulations based on ex vivo tissue biomechanics [20]. However, these studies rely on static optical coherence tomography (OCT) analyses, which fail to capture the dynamic, pulsatile nature of the tissues [6,8].

The initial investigation of pulsations in and around the optic nerve head (ONH) focused on the presence of venous pulsation. Recent studies have witnessed a surge in efforts to analyze tissue perfusion using OCT-angiography (OCT-A) and Doppler technologies [21,22,23]. Notably, low ONH perfusion has been linked to a higher prevalence and progression of glaucoma, even in the absence of elevated intraocular pressure (IOP) [24,25]. However, the current understanding remains limited regarding whether ONH pulsation reflects an imbalance between the forces exerted by blood flow, IOP, choroidal pulsation, and intraorbital pressure, or if it contributes to the initial pathophysiology of glaucoma.

Recent advances in optical coherence tomography (OCT) have led to the development of OCT elastography, a technique capable of quantifying the biomechanical properties of tissues in vivo [26], and it has been proven to facilitate assessment of biomechanical properties of multiple ocular structures and detect changes in biomechanical properties associated with changes in IOP [27]. However, despite the multiple applications of this technology it requires additional equipment, and it is not available in all clinical settings.

We developed a method to measure ONH changes during the peripheral pulse which on its own may have important implications [28,29,30]. It is possible that what we learn from these pulsatile measurements may lead to a better understanding of the pathophysiology of glaucoma and help with diagnosis and management. Examining the change in these pulsatile displacements when the IOP is reduced is one of the first steps to understanding their significance. The stress caused by IOP, peripapillary sclera stiffness, blood flow, and changes in these factors have a potentially detrimental impact on axon bundles and supporting cells in the ONH, particularly around the lamina cribrosa [29,31,32,33,34].

Each cardiac cycle influences ocular structures through a propagated blood pressure wave, leading to variations in choroidal volume, pulsatile tissue movements, and intraocular pressure. Since the eye is a dynamic and non-rigid organ, it exhibits complex responses to these hemodynamic and biomechanical forces, which are not yet fully understood due to the intricate anatomy of the eye. Similarly, research in neurology has shown that pulsatile movements of cerebrospinal fluid, driven by cardiac blood flow, significantly affect neural tissues and contribute to conditions like Chiari malformation [35]. This suggests that similar mechanisms could potentially impact the eye, given its direct connection to the brain and exposure to systemic pulsatile forces. 

Ocular pulse amplitude has also been correlated to changes in intraocular pressure due to glaucoma treatment [36,37]. It has been documented that large IOP decrease following trabeculectomy causes a decrease in OPA and choroidal thickening. Choroidal perfusion changes have also been reported in animal models after IOP manipulation [38], which could be indicative of the importance of the hemodynamic forces in the eye.

By measuring how these forces affect the ONH, researchers can better understand the physiological and pathological responses of the eye, potentially leading to breakthroughs in diagnosing and treating ocular diseases. Moreover, this understanding could lead to insights into how repetitive micro-stretching from pulsatile forces may contribute to tissue damage and disease progression, similar to observations made in certain brain conditions.

While current evidence does not establish a clear link between pulsatile displacement and ocular disease or visual outcomes, a deeper physiological and pathological understanding of this factor could justify its inclusion in future studies. 

## 2. Materials and Methods

The institutional review board of the Maisonneuve-Rosemont Hospital approved this study, which was conducted in accordance with the 1964 Declaration of Helsinki and its amendments. Written informed consent was obtained from all participants.

### 2.1. Patient Recruitment and Clinical Examination

Patients with POAG were recruited from the ophthalmology clinic at the Maisonneuve-Rosemont Hospital and were only included in the study if they were beginning topical drugs to reduce intraocular pressure, were selected for selective laser trabeculoplasty (SLT), or were scheduled to undergo filtration surgery, without the use of a drainage device, as part of their clinical care. Only one eye was included per participant, and a minimum IOP reduction of 3 mmHg was required. No additional procedures were performed as part of the study. 

A control group, defined as eyes with no IOP reduction intervention, of 13 subjects was recruited. Exams were performed within 4–6 weeks apart in the same anatomical position, using the built in OCT eye tracker.

Eye examinations and imaging were performed by the same ophthalmologists (ML and MMS, respectively). The visual function was assessed using a Zeiss Humphrey Field Analyzer Visual Field (Carl Zeiss Meditec, Dublin, CA, USA), and static OCT measurements were acquired using a Spectralis OCT (Heidelberg, Germany), as required by the clinical protocol.

The PlexElite 9000 OCT (Zeiss, Dublin, CA, USA) was used to acquire videos with an A-scan rate of 100 kHz, with a center wavelength of 1040 nm. The OCT device has an axial optical resolution of 6.3 microns and a transverse resolution of 20 microns. Images were acquired with no pupillary dilation in dim light conditions. 

### 2.2. Image Processing

The algorithm to generate the displacement maps was validated and described in detail in our previously published paper [30]. Briefly, we measured the displacement of ONH tissue caused by pulsatile blood flow changes over a period of 30 s (3000 images) using swept-source OCT. 

**Figure 1:**
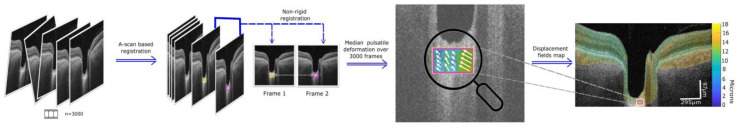
Pipeline to process data. A 3000-frame OCT video is acquired. Images are aligned by a global and a finer A-scan-based rigid registration. Non rigid registration is applied to obtain displacement fields that describe tissue changes between every pair of images. Displacement vectors are calculated for every pair of images, and the median absolute value of this field is calculated for the whole video. Finally, a graphical representation of the local changes in the median pulsatile displacement on one B-scan is obtained.

To minimize movement artifacts due to respiration, head movements, and saccades, consecutive images were rigidly registered, by applying global transverse and axial translations relative to a reference frame (Figure 1). To correct for finer motion artifacts and eye rotations, cross-correlation was computed between all A-scans of an image and those of the reference frame. Next, a linear fit of the maximum cross-correlation for all A-scans was used to calculate the axial translations that compensate rotation.

Once images were globally aligned and A-scan registration was performed, median pulsatile displacement fields were calculated between pairs of frames using the demons algorithm [39,40,41,42,43] and movement heatmaps were constructed from the median displacement of all the image pairs along the movie (Figure 1). The algorithm’s outcomes were validated previously to assess its response to different amplitudes, noise levels, and ability to identify physiological changes [30]. We applied this method to compare displacement maps of glaucoma participants imaged within 7 days before and 4–6 weeks after the clinical intervention. Participants with difficulty fixating or unclear media were excluded from the study.

**Figure 2:**
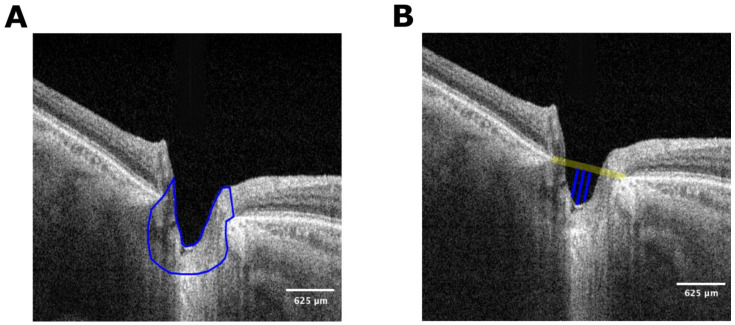
(**A**) Segmented optic nerve head. The highlighted region represents the section used for the analysis. The ONH area was manually traced by the same observer in all cases, using the Bruch’s membrane openings as references. The included area was the tissue in between both landmarks, the vitreous interphase, and the posterior border of the LC. The area between the internal limiting membrane and retinal pigment epithelium/Bruch’s membrane complex was manually delimited in both nasal and temporal retinas. (**B**) Pre-laminar tissue depth (“cup depth”) is defined by the maximum depth between a horizontal line connecting the nasal and temporal BMO and the deepest anterior lamina cribrosa and prelaminar tissue area. Two additional lines were traced 50 microns nasally and temporally to this central line and the total depth was defined as the average of the three lines.

The same observer (MMS) manually traced the ONH area in all cases and the Bruch’s membrane openings (BMO) as references (Figure 2A). The area between the internal limiting membrane and the RPE/Bruch’s membrane complex was manually delimited in both the nasal and temporal retina. The lamina cribrosa and prelaminar tissue depth were measured using the BMO plane to the deepest level of the cup (the most anterior portion of the prelaminar tissue) [44]. First, a line was drawn at the maximum point, and then two other lines were traced, one nasal and the other temporal, 100 microns from this line. The mean distance of these three measurements was defined as the anterior prelaminar tissue depth and lamina cribrosa depth (Figure 2B).

Peripapillary choroid thickness was measured in the nasal and temporal choroid 100 microns from the BMO for each one of the B-scans.

### 2.3. Statistics

We performed statistical analysis using R programming software (version 2022.12.0+353). To generate the plots, we used the ggstatsplot package [45]. Descriptive statistics were applied to the demographic data, while the Student *t*-test, Wilcoxon (in non-parametric distribution data), and ANOVA were used for continuous and categorical variables. Multivariate analysis was conducted to determine the correlation between pulsatile optic nerve displacement, demographic, and clinical data.

Non-parametric paired tests (Wilcoxon) were used to compare the main clinical parameters extracted for the Spectralis OCT, which was used to obtain the standard of care OCT parameters.

## 3. Results

### 3.1. Demographic and Clinical Data

Initially, 53 patients were included in the study; however, due to excessive movement, media opacities or post-operative complications, a total of 41 participants with POAG were finally included in the study. Within this cohort, 9 subjects were also diagnosed with axial myopia (axial length > 25 mm), 19 with no myopia and 13 corresponded to the no-intervention control group. The demographic and clinical characteristics of the intervention groups are listed in Table 1.

The mean age was 68 years for the glaucoma group, 64 years for the glaucoma and axial myopia one and 66 for the control, with a sex distribution of 73%, 44%, and 46% of female participants in each cohort, respectively. There was a significant intraocular pressure reduction for both treatment cohorts (9.1 ± 7 mmHg and 6.2 ± 4 mmHg). Images were taken between 4 and 6 weeks after intervention.

For the intervention groups, no difference was found in the visual field before and after the treatment. Overall, there was no significant difference between the main clinically used parameters (RNFL thickness, BMO area, and ganglion cell complex (GCC) volume) between different axial length groups at baseline. Following intervention, there was a significant decrease in the inferior retinal fiber layer thickness (RNFL) in both groups (*p* = 0.05 for both the AL < 25 mm and AL ≥ 25 mm) and in the GCC volume in the non-myopic glaucoma cohort (*p* = 0.02).

There was no significant difference between the peripapillary choroidal cross-sectional thickness area on the B-scans before and after intervention, whereas the anterior prelaminar tissue depth change was only significant in the non-myopic glaucoma cohort. It changed from a mean depth of 1033 ± 600 μm to 973 ± 559 μm.

### 3.2. Pulsatile Displacement Change 

**Figure 3:**
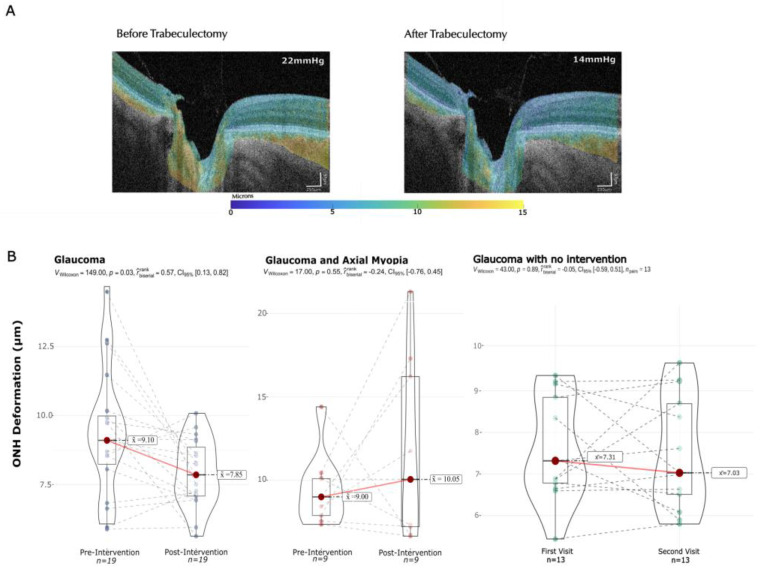
Median pulsatile displacement changes before and after IOP decrease. Panel (**A**): Example of pulsatile displacement maps corresponding to a subject before and after trabeculectomy with an IOP decrease of 8 mmHg. Panel (**B**): Median pulsatile displacement changes in the ONH in glaucoma, glaucoma plus myopia and control cohorts.

The main, and most interesting, outcome of our results is the measurement of the median pulsatile displacement in the ONH as shown in Figure 3. 

An example of the changes in the median pulsatile displacement map is depicted in panel A. The ONH is delimited between the two Bruch’s membrane openings and the posterior surface of the prelaminar tissue (see Figure 3).

Before the intervention, there was a median pulsatile displacement of 9.1 ± 2 μm compared to a displacement of 7.9 ± 1 μm after IOP decrease (*p* = 0.03) in the non-myopic glaucoma cohort, resulting in a significant 13.7% decrease in pulsatile displacement after the intervention compared to the baseline. In contrast, in the myopic glaucoma cohort, the baseline displacement was 9.0 ± 1 μm compared to 10.1 ± 2 μm after the intervention resulting in a 10.9% increase from the baseline; notably this effect is not statistically significant (*p* = 0.55) (Figure 3). 

In the control group there was a baseline displacement of 7.31 ± 2 μm and a mean displacement of 7.03 ± 2 μm in the second visit (*p* = 0.89). 

Regarding the directionality of the change, while most subjects exhibited a reduction in displacement, it is noteworthy that in the normal AL and glaucoma group, six patients (31.6%) experienced an increase in displacement after the intervention, in contrast to four patients in the glaucoma and axial myopia group (44.4%).

The magnitude of the change in median pulsatile (before–after IOP reduction) displacement (in microns) was used as an outcome to explore the correlation with the demographic and clinical variables. The multivariate analysis showed no significant association between changes in pulsatile displacement and age, sex, IOP change or glaucoma severity in the complete cohort or the AL < 25 and AL ≥ 25 mm groups separately. The type of treatment (surgical versus medical) was also not found to be an influential factor in the displacement change (R = 0.02, *p* = 0.5).

**Figure 4:**
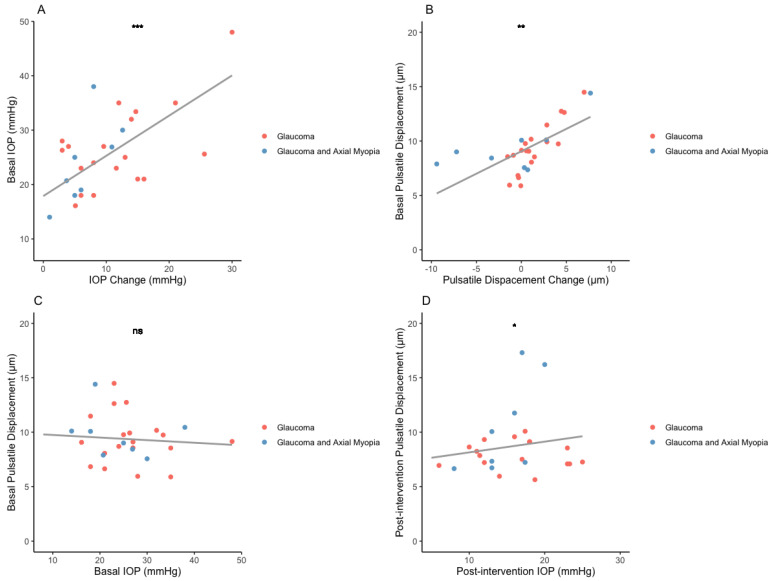
Correlation between intraocular pressure and pulsatile deformation. (**A**) Basal IOP correlation with absolute IOP change (mmHg). (**B**) Basal pulsatile deformation correlation with absolute displacement change (μm). (**C**) Relationship between IOP and deformation before intervention. (**D**). Relationship between IOP and deformation after intervention.

A detailed analysis was conducted to investigate the correlation between IOP and pulsatile displacement. As discussed, the IOP change did not exhibit a significant correlation with the change in pulsatile displacement (R = 0.01, *p* = 0.2). Interestingly, a positive correlation was observed between basal IOP and the IOP change in mmHg (R = 0.4, *p* = 0.001, CI [0.3–0.8]) (Figure 4A), and a similar correlation was identified between basal pulsatile displacement and the displacement change (R = 0.2, *p* = 0.004, CI [0.1–0.6]) (Figure 4B). This implies that higher basal values (before intervention) of IOP and pulsatile displacements are correlated with a larger change, respectively, after the intervention.

Moreover, when we analyzed the relationship between IOP and pulsatile displacement at baseline, there was no significant correlation before treatment (R = 0.03, *p* = 0.6). However, this changed when we examined the postoperative data. We observed a significant correlation between IOP and pulsatile displacement after the intervention (R = 0.2, *p* = 0.02, CI [0.04–0.5]) (Figure 4C,D).

Additionally, a modest positive correlation was found between the absolute displacement change and the change (before–after IOP reduction) in the anterior prelaminar tissue depth upon intervention (R = 0.2, *p* = 0.03, [CI 0.0009–0.02]) in the complete cohort. This multivariate analysis was performed using age, IOP and basal prelaminar tissue depth as confounders.

Given the difference between IOP decrease in both cohorts, the absolute IOP change was used as a confounder in the multivariate analysis, along with axial length, and no significant effect was found in the degree of IOP-reduction on pulsatile displacement in our data. For intraocular pressure change there was no correlation with the amount of pulsatile displacement change (R = 0.009, *p* = 0.9).

No significant correlation was found between any of the OCT and VF field parameters described in Table 1 and the pulsatile displacement at baseline.

## 4. Discussion and Conclusions

Our study found that a reduction in IOP leads to a significant decrease in pulsatile displacement of the ONH in patients with POAG without myopia. In contrast, the myopic group showed a non-significant increase in pulsatile displacement after IOP reduction, despite having a smaller IOP reduction and a 10% greater rate of surgical intervention. Since more subjects in the myopic cohort had an increase of the pulsatile displacement and given the sample size, a bigger longitudinal study on axial myopia patients would help understand the difference in the biomechanical response in this specific group of patients.

There was no ONH pulsatile displacement when the IOP values remain constant, as shown in the control group which suggest that the biomechanical response may be related with the IOP levels in glaucoma patients.

The results indicate that IOP has an impact on the pulsatile displacement of the ONH, and the relationship between these two factors may be affected by myopia. The difference between the myopic and non-myopic groups could be attributed to the fact that myopic eyes tend to have less stiff tissue compared to non-myopic eyes [46,47,48]. However, our sample size in the myopic group was too small to be certain that a significant difference existed in the behavior of the two groups.

The displacement observed in this study is most likely the result of the combination of pulsatile changes occurring in the eye, especially the pulsations of the choroidal and retinal vasculature and the pulsatile aqueous outflow, which intersect with the mechanical properties of the ocular tissues. In vitro studies on human tissue have shown that cyclical mechanical stretch at the heart rate frequency can affect extracellular matrix transcription genes in the lamina cribrosa, as suggested by Kirwan et al. [49]. This molecular effect could potentially explain the neuroprotective effect of IOP from a biomechanical perspective.

Jin et al. added relevant evidence using finite element modelling, highlighting the importance of the vascular component, studied in silico. The models indicate that during the cardiac cycle, the ocular pulse amplitude and choroidal expansion can deform the ONH with a net shearing of neural tissues within the neuroretinal rim [50]. Here we presented the clinical applications of this novel method to measure the ONH tissue displacement driven by the ocular pulsations and choroid which we have previously hypothesized and measured [29,51].

The pulsation of the ocular tissue is a result of a complex interplay of mechanical forces acting around the ONH. These forces include the response to changes in IOP, intracranial pressure [17], choroidal perfusion [52], retinal perfusion, and the biomechanical properties of the ONH tissue, and other ocular tissues such as ocular rigidity [53,54]. Our findings suggest a significant decrease in pulsatile displacement of the ONH after IOP reduction in patients with POAG without myopia, even after correcting for baseline IOP and IOP change. This suggests that while there may be a physiological interaction between IOP and tissue response, the strain of the tissue is more closely linked to the biomechanical status of the tissue (in this case seen as the lamina cribrosa position change) than to the IOP change itself. This notion needs to be examined in a much larger study.

The current evidence on biomechanical modeling of the human eye mainly focuses on static OCT imaging. By analyzing video OCT, instead of a static assessment, we are able to capture the pulsatile changes along with the influence of IOP. This feature may permit us to better explore biomechanics-based risk factors. However, it also adds the constraint of analyzing a smaller anatomical area compared to other studies [2,17,50,55]. The pulsatile displacement may in future be used to estimate the mechanical properties of the ONH, without resorting to manipulating the IOP.

We conducted a thorough analysis of static parameters in standard OCT imaging and visual field, taking into account factors such as IOP, type of intervention, and demographic variables that may influence the anatomical characteristics of the lamina cribrosa, such as age [56] (see Table 1). Our results revealed a modest correlation between the change in position of the anterior surface of the prelaminar tissue following IOP reduction and the change in pulsatile displacement following intervention in both myopic and non-myopic patients. This finding supports the idea that changes in tissue may influence the response of the lamina cribrosa to cyclic cardiac inputs [20,50].

Another potential limitation is that the observed differences in pulsatile displacement response to IOP reduction between myopic and non-myopic patients with primary open-angle glaucoma (POAG) need to be confirmed in a larger cohort that is also better matched for the degree of IOP reduction. If confirmed, the differences observed between myopic and non-myopic eyes could potentially be attributed to the underlying mechanical properties of the ONH. In non-myopic eyes with glaucoma, a thinner choroid has been reported compared to normal population [12,52,57], and this could result in reduced choroidal pulsatility which may be the primary driving force for lower ONH pulsatility when IOP is also lowered. On the other hand, in myopic eyes the choroid is even thinner, as is the lamina cribrosa, and the sclera is thinner and less rigid compared to non-glaucomatous eyes [58]. Mechanical properties of the sclera may be the dominant factor in myopia, allowing for more movement with the pulse as IOP is reduced. This suggests that there is a complex interplay between factors such as IOP, choroidal pulsatility, and scleral mechanics that may be modified in the presence of myopia. While it is still early to support this theory, the application of this new tool may provide a starting point to explore the role of these vascular forces within a clinical context.

Preoperative intraocular pressure has been identified as a predictor of IOP reduction following glaucoma treatment and cataract surgery [59,60,61]. Our data support this evidence. We demonstrate that a higher basal IOP is correlated with a more substantial IOP reduction. Similarly, the basal pulsatile displacement is correlated with a greater change in displacement as part of the response to treatment. This correlation is independent of the absolute value of IOP reduction, which could provide additional information regarding the treatment response.

Another noteworthy finding is the increased correlation between IOP and pulsatile deformation, as illustrated in Figure 4. A similar change in this correlation has been described in the cornea, particularly corneal hysteresis, where authors observed an increased correlation between the two variables in weeks 2 and 4 after IOP reduction. In this case the recovery is suggested to be related to microstructural changes that affect CH rather than the macrostructural changes, evidence that has also been presented in animal models [62].

While the specific correlation between IOP and pulsatile deformation measured by OCT is not widely reported in the literature, there is growing evidence describing the remodeling of the ONH tissue in response to treatment [63,64] and in some cases it has been clinically described that RNFL thickness can improve after IOP has been stabilized [65,66]. Our method is a novel tool that, applied to a bigger cohort, could lead to deeper analysis on the correlation of biomechanics and positive outcomes to treatment in glaucoma.

When IOP was reduced by surgery, our second imaging session was conducted after 6 weeks because earlier imaging may have shown lower quality. When medical therapy was used to lower IOP, imaging was performed after the documented IOP drop, which was within 4–6 weeks. By not imaging immediately after an acute change, we are allowing some time for tissue remodeling to occur [67,68], which can be challenging to account for when analyzing our results. Prospective data are needed to better understand the role of tissue remodeling in glaucoma progression and its correlation with pulsatile displacement.

The morphological differences at the ONH in glaucoma patients with and without myopia pose a continuing challenge for diagnostics and follow up. The morphological characteristics of the ONH exhibit high variability, making it difficult to establish a standard set of parameters for diagnosis and monitoring, especially in myopia [69]. Variations in optic disc size, optic nerve fiber density, lamina cribrosa pores, interpore connective tissue area, and cilioretinal artery frequency also contribute to the complexity of the problem [11]. It is possible that the pulsatile displacement of the ONH could be a new parameter that has some value in diagnosis and follow up of POAG, in addition to its potential role in the pathophysiology of the disease. Further studies will be required.

There is a notable variation of the displacement value among individuals, as seen in Figure 3, which could be explained by the multifactorial nature of this biomarker. There is considerable variation within individuals of tissue rigidity, intralaminar pressure and response to intraocular pressure treatment. While the response remains fairly consistent within the normal AL and glaucoma group, a careful interpretation of the results, including the consideration of multiple clinical variables, should be performed in future studies.

The neuroprotective effect of IOP decrease also exhibits significant variability within individuals [70], making it challenging to rely solely on IOP measurements for clinical monitoring of the ONH. We speculate that the biomarker of pulsatile ONH displacement could become complementary to IOP itself as a tool for clinical assessment upon further investigation. Larger cross-sectional and prospective studies will be required.

## Figures and Tables

**Table 1:** Demographic and clinical characteristics of included study participants.

Parameter	Group (n = 28)						
	Glaucoma (n = 19)			Glaucoma and Myopia (n = 9)			
	Pre-Intervention	Post-Intervention	*p*-Value *	Pre-Intervention	Post-Intervention	*p*-Value *	Baseline Comparison (*p*-Value)
Age (years)	67.7 ± 11		-	64.4 ± 14		-	0.4 ^+^
Sex (female %)	14 (73%)		-	4 (44%)		-	0.1 ^ο^
Intervention (surgical %) **	8 (50%)		-	4 (44%)		-	-
AL (mm)	23.19 ± 0.7		-	25.89 ± 0.5		-	<0.005 ^+^
IOP (mmHg)	25.65 ± 8	16.5 ± 7	<0.005	22.2 ± 6	16.0 ± 9	0.01	0.2 ^+^
IOP change (mmHg)	9.1 ± 7		-	6.2 ± 4		-	0.01 ^+^
Glaucoma severity ***							
Early (n (%))	7 (37%)		-	4 (44%)		-	-
Moderate (n (%))	6 (31.5%)		-	2 (28%)		-	-
Severe (n (%))	6 (31.5%)		-	3 (28%)		-	-
Anatomical and Functional Assessment							
Visual Field MD (dB)	−3.67 ± 6	−3.68 ± 6	0.4	−5.51 ± 3	−5.40 ± 4	0.8	0.5 ^+^
BMO area (μm^2^)	1.89 ± 0.4	1.88 ± 0.4	0.7	2.26 ± 0.5	2.25 ± 0.5	0.1	0.4 ^+^
GCC volume (mm^3^)	0.91 ± 0.1	0.88 ± 0.2	0.02	0.86 ± 0.1	0.87 ± 0.1	0.7	0.6 ^+^
Peripapillary CT area (mm^2^)	974 ± 353	1140 ± 380	0.1	499 ± 369	661 ± 352	0.7	<0.005 ^+^
Anterior PLT depth (μm)	1033.02 ± 600	973.39 ± 559	0.05	753.46 ± 439	740.12 ± 452	0.5	0.3 ^+^
RNFL Thickness (μm)							
Superior	96 ± 26	94 ± 24	0.08	96 ± 16	92 ± 17	0.01	0.5 ^+^
Inferior	94 ± 33	87 ± 35	0.05	101 ± 36	89 ± 32	0.01	0.8 ^+^
Temporal	57 ± 15	55 ± 13	0.7	63 ± 11	64 ± 13	0.3	0.4 ^+^
Nasal	59 ± 16	61 ± 16	0.1	56 ± 16	61 ± 17	0.5	0.2 ^+^

* Paired Wilcoxon test. ** Compared to medical intervention with eyedrops or SLT (n (%)). *** Classification based on Hodapp–Anderson–Parrish criteria. IOP (intraocular pressure), AL (axial length), RNFL (retinal nerve fiber layer), BMO (Bruch’s membrane opening), GCC (ganglion cell complex), CT (choroid thickness), PLT (prelaminar tissue). ^+^ Welch’s *t* test, ^ο^ Chi-Square Test.

## Data Availability

The data is not publicly available due patient privacy and ethical committee regulations. Restrictions apply to the availability of these data.
